# Left ventricular unloading with gentle chest compressions for patients on veno-arterial extracorporeal membrane oxygenation: two case reports

**DOI:** 10.3389/fcvm.2024.1435935

**Published:** 2024-07-29

**Authors:** Lingyu Jiang, Minyan Huang, Shulin Xiang, Bin Xiong, Guibin Li, Yonglong Zhong, Lin Han

**Affiliations:** ^1^ Department of Intensive Care Unit, People’s Hospital of Guangxi Zhuang Autonomous Region, Guangxi Academy of Medical Sciences, Nanning, Guangxi Zhuang Autonomous Region, China; ^2^ Department of Thoracic Surgery, the People’s Hospital of Guangxi Zhuang Autonomous Region, Guangxi Academy of Medical Sciences, Nanning, Guangxi Zhuang Autonomous Region, China

**Keywords:** gentle chest compression, VA-ECMO, left ventricular unloading, heart failure, pulsatile contraction

## Abstract

Insufficient ventricular unloading is a serious complication during veno-arterial extracorporeal membrane oxygenation (VA-ECMO) that has a crucial impact on patient outcomes. The existing conservative treatment options are limited, while mechanical decompression techniques are challenging and restricted in terms of their adoption and application. Two patients with cardiogenic shock experienced insufficient left ventricular unloading with no pulsatile contraction and aortic valve closure during VA-ECMO support. Gentle chest compression was applied to establish an active left ventricular drainage mechanism, which prevented the formation of intracardiac thrombi. No life-threatening complications or technical problems occurred. Therefore, gentle chest compression was established as an effective and safe method for treating insufficient left ventricular unloading in VA-ECMO patients.

## Introduction

Veno-arterial extracorporeal membrane oxygenation (VA-ECMO) is an advanced extracorporeal life support method that temporarily replaces the patient's heart pump function, allowing time for recovery of the damaged myocardium (1, 2). However, the arterial end of VA-ECMO typically involves cannulation via the femoral artery, which opposes the natural flow direction in the aorta. This reversed blood flow perfusion subsequently increases the heart's afterload, hindering the ejection of blood from the left ventricle. Consequently, this leads to an increase in left ventricular end-diastolic pressure, dilation of the left ventricle, and augmented stress on its wall. This condition is referred to as insufficient left ventricular unloading (3), which presents additional challenges for patients with cardiogenic shock (4) and might have a deleterious long-term effect (5, 6).

The current prevailing trend suggests that earlier or prophylactic combined left ventricular unloading is likely to yield greater benefits (5, 7). In recent years, mechanical left ventricular decompression methods have been widely used. These include left ventricular assist devices, such as an intra-aortic balloon pump, direct decompression through trans-thoracic left atrial and left ventricular catheterization, percutaneous trans-aortic valve left ventricular decompression, and other techniques (8, 9). Currently, there is no consensus on which unloading strategy should be adopted, with 65% of studies citing the risk of complications as the primary reason for not using mechanical left ventricular unloading (10). Additionally, mechanical decompression methods have extremely high requirements for patient selection, surgical techniques, and management. The present report described two cases where a gentle method of chest compressions was used as a replacement technique to decompress the left ventricle in VA-ECMO patients and resulted in successful resuscitation.

## Case presentation

### Patient 1

The first case describes a 39-year-old man who spent one day at a small hospital due to acute extensive myocardial infarction. ECMO organ support was indicated in the case of no improvement with routine treatment. During the ECMO treatment, the patient repeatedly experienced loss of arterial pulsation accompanied by a pulse pressure difference of <10–15 mmHg. Large amounts of watery secretions emerged from the artificial airway. Portable echocardiography revealed that the aortic valve was unable to open, the left ventricle was in a creeping state, and the right ventricular contractile function remained satisfactory. Pulmonary ultrasonography showed bilateral diffuse B3 lines. Based on these observations, a comprehensive diagnosis of excessive left ventricular loading was ultimately made.

**Figure 1:**
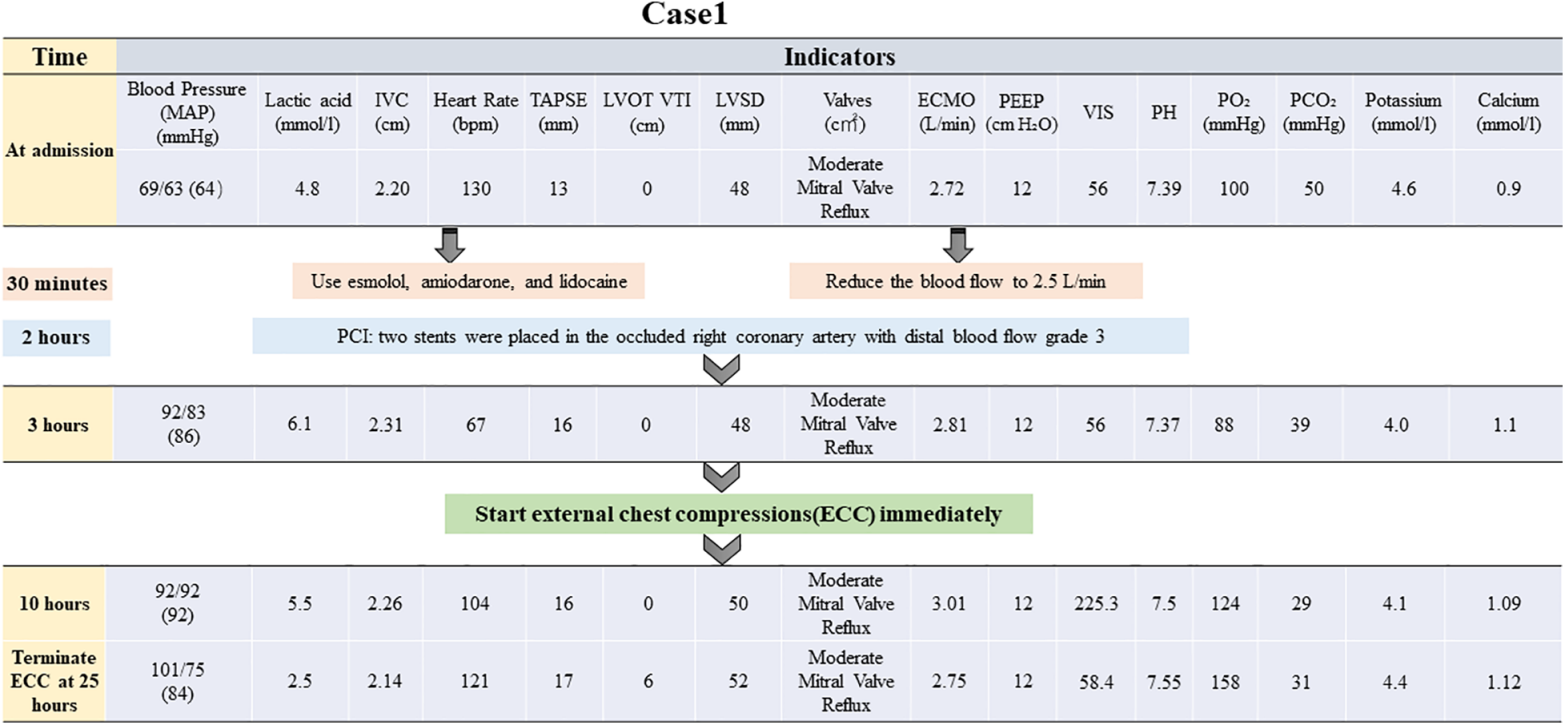
Brief timeline flowchart for patient treatment during chest compression (Case 1). MAP, mean arterial pressure; IVC, inferior vena cava; TAPSE, tricuspid annular plane systolic excursion; ECMO, extracorporeal membrane oxygenation; PEEP, positive end-expiratory pressure; PO₂, partial pressure of oxygen; LVOT VTI, left ventricular outflow tract velocity time integral; LVID, left ventricular internal diameter; VIS, vasoactive-inotropic score; PCI, percutaneous coronary intervention.

**Figure 2:**
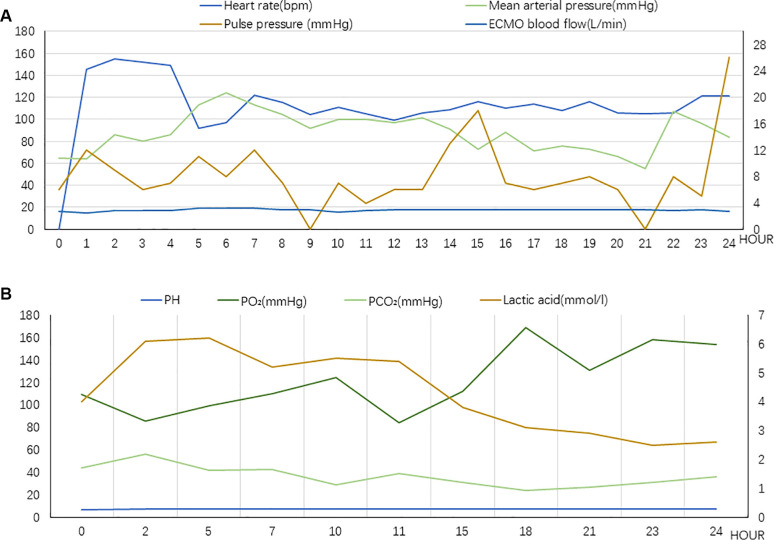
(**A**) Vital signs during chest compression (Case 1). The value of Pulse pressure and ECMO blood flow correspond to the secondary axis. (**B**) Laboratory tests indicators during chest compression (Case 1). The value of lactic acid corresponds to the secondary axis.

At this juncture, an emergency percutaneous coronary intervention was promptly initiated to urgently reopen the right coronary artery and reestablish blood flow to the affected areas. Concurrently, measures to reduce the load on the left ventricle were implemented, including managing arrhythmias, administering dobutamine to strengthen myocardial contractility, and prescribing furosemide for diuretic therapy lasting for 2 h. None of these treatments were effective. In the absence of mechanical means for left ventricular decompression, chest compressions were used as an emergency measure and administered intermittently at a frequency of 5 min/h, with a depth of 5 cm, and a rate of 100 bpm. The patient's blood pressure began to fluctuate after 25 h of intermittent chest compressions supported by ECMO. A flowchart summarizing the treatment process of ECMO and intermittent chest compressions is presented in Figure 1. The improvement in all indicators led to the cessation of chest compressions (Figure 2).

After withdrawing ECMO support, the echocardiography results revealed the formation of a left ventricular apical aneurysm accompanied by a mural thrombus measuring 33 mm × 9 mm. As a result, nadroparin calcium was prescribed for anticoagulation therapy and was subsequently replaced with warfarin treatment for three months. In summary, the patient received ECMO support for nine days and ventilator assistance for 13 days and remained at the intensive care unit (ICU) for 17 days. The total hospital stays lasted for 24 days. A follow-up examination 6 months later demonstrated that the patient recovered to an excellent state of health, retained full capacity for independent living, and displayed no residual signs of organ dysfunction.

### Patient 2

A 50-year-old male patient with no prior significant medical history presented with symptoms of chest tightness and chest pain following an upper respiratory tract infection and was diagnosed with fulminant myocarditis. The patient's right radial artery exhibited no palpable pulsation during ECMO-assisted circulation support, leading to a pulse pressure difference of zero. Notably, large quantities of watery secretions emerging from the artificial airway were observed. Concurrent bedside echocardiography examination revealed that the aortic valve failed to open, with the left ventricle displaying a creeping motion and the right ventricle showing decreased contractile function. Furthermore, diffuse B3 lines were visible in both lung fields. After a thorough evaluation, it was determined that there was inadequate unloading of the left ventricle.

**Figure 3:**
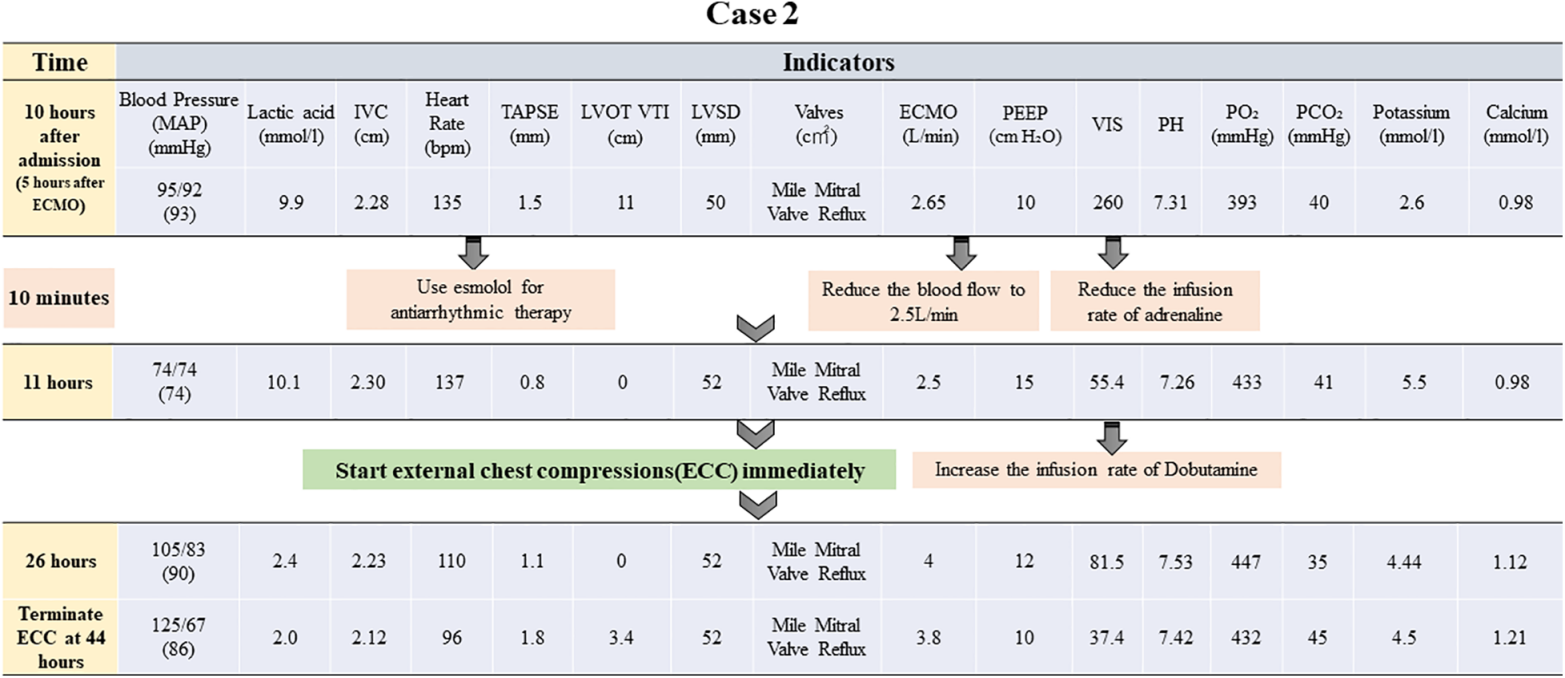
Brief timeline flowchart for patient treatment during chest compression (Case 2). MAP: mean arterial pressure, IVC: inferior vena cava, TAPSE: tricuspid annular plane systolic excursion, ECMO: extracorporeal membrane oxygenation, PEEP: positive end-expiratory pressure, PO_2_: partial pressure of oxygen, LVOT VTI: left ventricular outflow tract velocity time integral, LVID: left ventricular internal diameter, VIS: vasoactive-inotropic score.

**Figure 4:**
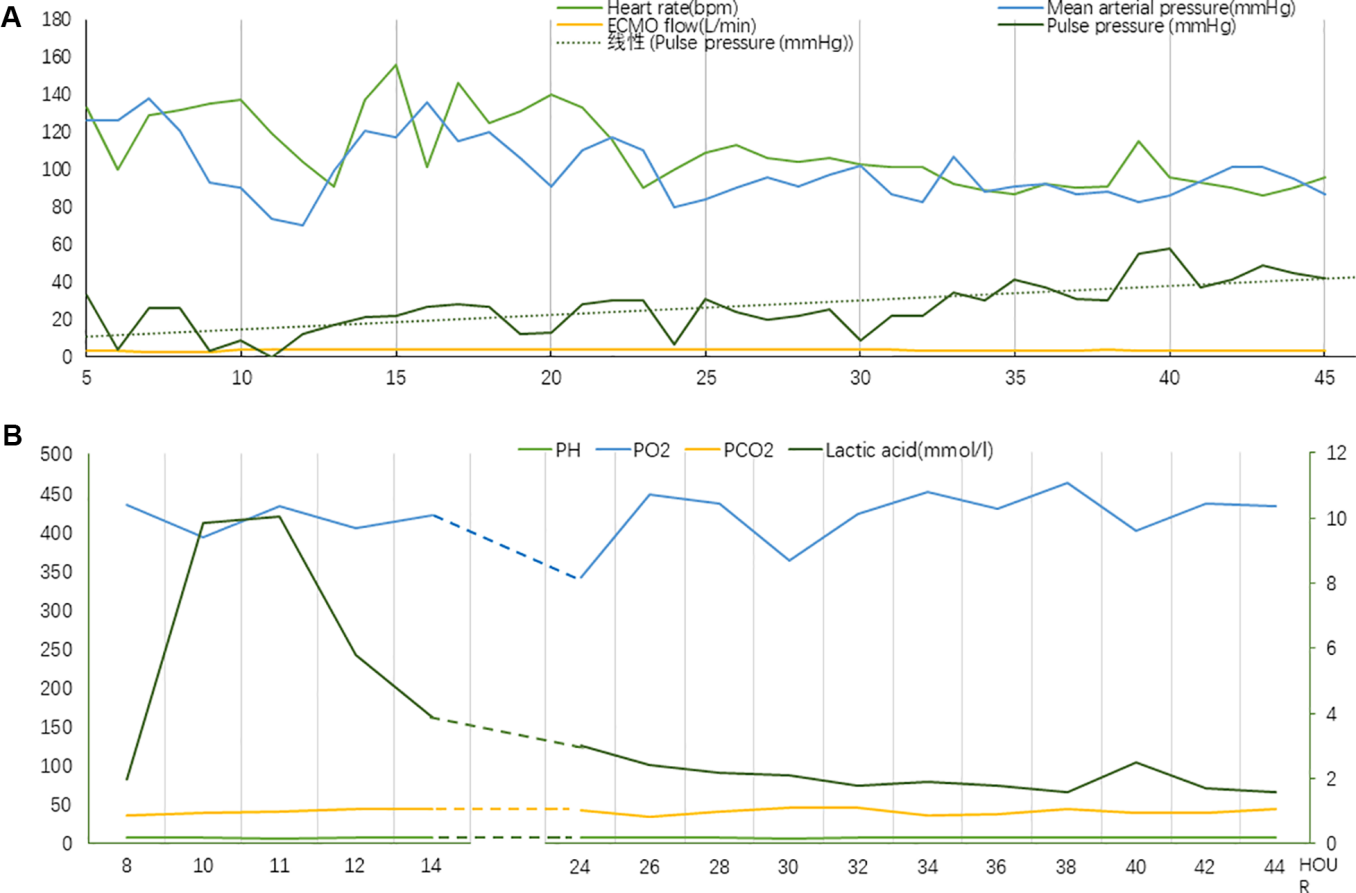
(**A**) Vital signs during chest compression (Case 2). (**B**) Laboratory tests indicators during chest compression (Case 2). The value of lactic acid corresponds to the secondary axis.

Prompt action to reduce the burden on the left heart was initiated by rapidly removing excess fluid using continuous renal replacement therapy (CRRT). Furthermore, dobutamine was prescribed to boost myocardial contractility, while beta-blockers were administered to regulate the heart rate. The patient exhibited signs of inadequate perfusion despite adjusting the ECMO flow rate to 2.65 L/min, with lactic acid levels continuously surging to reach a maximum of 9.9 mmol/L. The ECMO flow rate was subsequently increased once again to guarantee enough organ and tissue perfusion. Next, chest compressions were utilized as an emergency measure, maintaining a depth of 3 cm and a frequency of 80 bpm. After approximately 33 h of treatment, the patient's blood pressure began to fluctuate normally. A repeat bedside echocardiogram revealed that velocity time integral reached 3.4 cm and lactic acid level decreased to 1.6 mmol/L. Chest compressions were terminated following these improvements. A flowchart summarizing the treatment process and the important indicators were presented in Figures 3, 4.

One week later, a repeat echocardiogram demonstrated mild enlargement of the left ventricle, along with improved motion of the left ventricular wall and enhanced left ventricular contractile function, resulting in an ejection fraction of 38%. Overall, the patient underwent ECMO support for nine days, received ventilator assistance for 12 days, and stayed at the ICU for 16 days. The overall hospital stays lasted for 21 days. A 6-month follow-up examination confirmed the patient's good health status and absence of any residual organ dysfunction.

## Discussion

The present study described the use of gentle chest compression to mitigate insufficient left heart unloading during VA-ECMO treatment after unsatisfactory conventional treatments. Notably, both patients recovered successfully and achieved positive outcomes. Moreover, compromised ECMO efficiency reduced oxygen supply, leading to insufficient tissue perfusion. This observation is consistent with those reported in a study by Myneni et al. (11). These treatment approaches, including CRRT, dobutamine, beta-blockers, and reducing ECMO flow rate, may be unfavorable for patients with severe heart failure. In 1960, Kouwenhoven et al. (12) first proposed that chest compressions produce antegrade blood flow as a result of directly compressing the heart between the sternum and the spine, which is known as the cardiac pump theory. According to this theory, chest compressions can be a rescue measure to prevent left chamber thrombosis and improve pulmonary circulation during left ventricular distension syndrome, in which an active left ventricular drainage mechanism is established (13). The first patient in the present study received chest compressions with a frequency and depth described in the 2015 International Guidelines for Cardiopulmonary Resuscitation (14). With the support of ECMO, only 15 pulse pressure differences were needed. Due to chest compression depth influences haemodynamic parameters during cardiopulmonary resuscitation (15), the 3 cm depth was implement to achieve carotid blood flow in the second patient. This change relieved left ventricular overload and achieved the desired effect of forward pulsatile blood flow ranging from 15 to 40 mmHg. If the difference in pulse pressure remained above 15 mmHg for more than 30 min, it was defined as the presence of natural pulsatile blood flow and chest compression was stopped. According to a study comparing the complications associated with chest compression depths of <5 cm, 5–6 cm, and >6 cm, the rate of iatrogenic injuries gradually increased with respective rates of 28%, 27%, and 49% (16). Remarkably, both patients in the present study were spared from any complications associated with chest compression lasting for more than 24 h. In the first patient, an intraventricular thrombus was observed after ECMO withdrawal. According to prior reports, the incidence of left ventricular thrombus with left ventricular aneurysm can reach 42% (17). Therefore, considering that this patient developed a left ventricular apical aneurysm and that the intramural thrombus was attached to the left apical wall, it is possible that the intramural thrombus was a complication of myocardial infarction rather than a result of insufficient chest compression. Although the duration of myocardial stunning in the second patient was relatively long and lasted for 45 h, the patient's cardiac function ultimately recovered similar to the results reported in previous studies (18, 19). Thus, myocardial stunning likely occurred, because the myocardial enzyme spectrum remained relatively unchanged throughout the patient's illness. Both patients received CRRT for 24 h and were able to resume spontaneous urine output by the time of discharge.

Although the expected effect of the gentle chest compression technique on patients with insufficient left ventricular unloading under ECMO support in the present study was encouraging, there were still some shortcomings associated with this method. First, the compression standards were inconsistent, including intermittent and continuous methods with significant differences in depth and frequency. Second, there was a lack of dynamic monitoring of intracoronary blood supply within the heart, such as transesophageal echocardiography, which can simultaneously assess the pressure in each atrium and ventricle.

The gentle chest compression technique yielded promising outcomes, making it an alternative diagnostic and therapeutic avenue for managing insufficient left ventricular unloading in VA-ECMO patients. In the future, more studies will focus on the gentle chest compression technique in the context of left ventricular unloading, aiming to provide more evidence-based medical data for clinical diagnosis and treatment.

## Data Availability

The raw data supporting the conclusions of this article will be made available by the authors, without undue reservation.
