# Supplementary figures and images for: Left ventricular unloading with gentle chest compressions for patients on veno-arterial extracorporeal membrane oxygenation: two case reports
Source: Front Cardiovasc Med. 2024 Jul 29;11:1435935. doi: 10.3389/fcvm.2024.1435935 (PMC11318276; doi:10.3389/fcvm.2024.1435935)

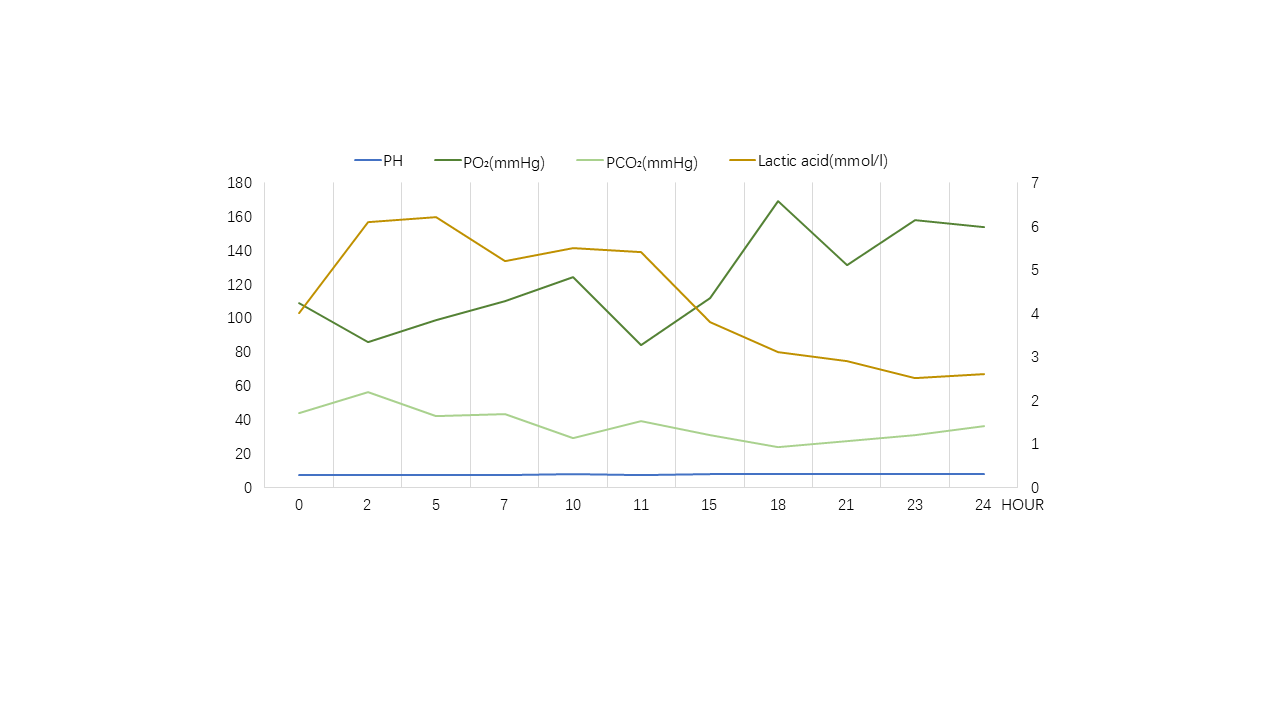

Supplement: Supplementary file 1 [file Image1.tif]

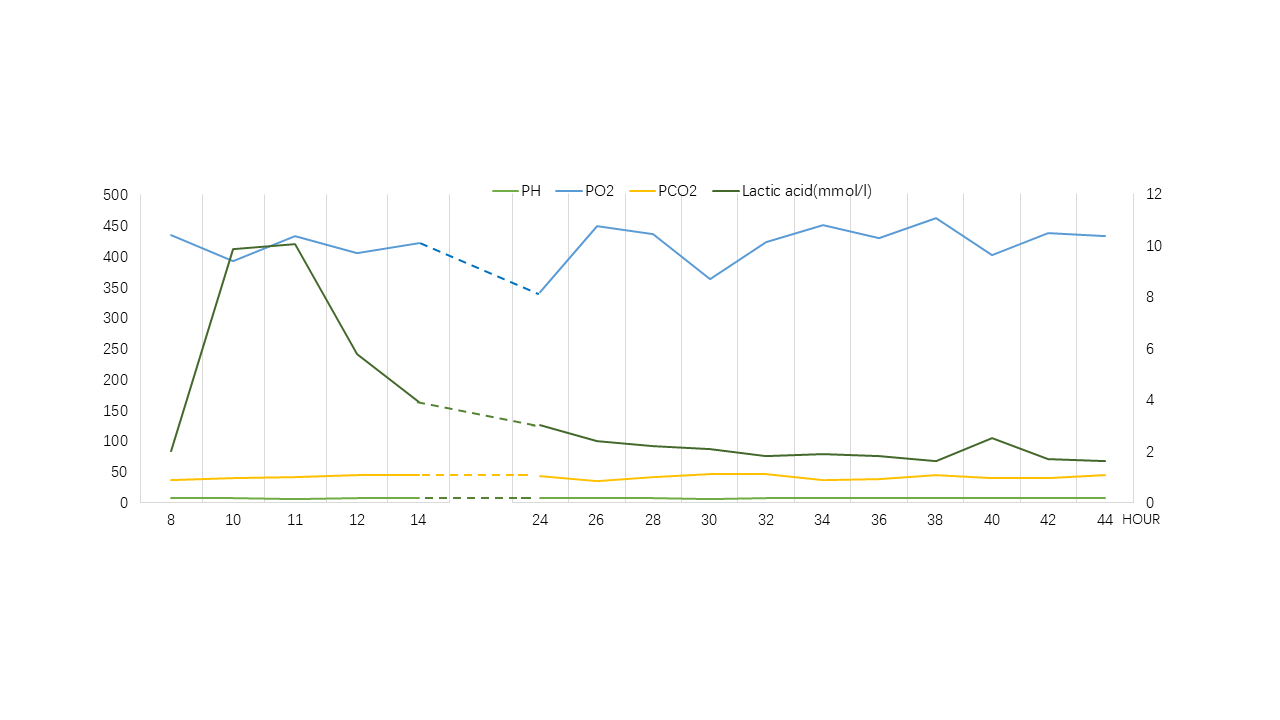

Supplement: Supplementary file 2 [file Image2.tif]
